# Study of the Microstructure and Ferroelectric and Magnetic Properties of Three-Component Multiferroic Composites

**DOI:** 10.3390/ma19204309

**Published:** 2026-10-13

**Authors:** Dariusz Bochenek, Dagmara Brzezińska, Artur Chrobak, Dariusz Oleszak, Piotr Duda

**Affiliations:** 1 Faculty of Science and Technology, Institute of Materials Engineering, University of Silesia in Katowice, 75 Pułku Piechoty 1a, 41-500 Chorzów, Poland; artur.chrobak@us.edu.pl; 2 Faculty of Materials Science and Engineering, Warsaw University of Technology, Wołoska 141 02-507, 02-507 Warsaw, Poland; dariusz.oleszak@pw.edu.pl; 3 Faculty of Science and Technology, Institute of Biomedical Engineering, University of Silesia in Katowice, Będzińska 39, 41-200 Sosnowiec, Poland; piotr.duda@us.edu.pl

**Keywords:** PZT-type ceramics, perovskite-type materials, ferrites, multiferroics, multiferroic composite materials

## Abstract

In this study, two ternary multiferroic composites (BT-PZT-F and BT-BF-F) with a ferroelectric phase (90%) and a magnetic phase (10%) were developed. In the case of the BT-PZT-F multiferroic composite, the ferroelectric components were BaTiO_3_ (BT) and PZT-type material (PZT) (in a 50:50 ratio), whereas the magnetic component was Ni_0.64_Zn_0.36_Fe_2_O_4_ ferrite (F). On the other hand, BaTiO_3_ (BT) and BiFeO_3_ (BF) (in a 50:50 ratio) were the ferroelectric components in the BT-BF-F multiferroic composite, whereas the magnetic component was ferrite F. The free sintering method has been applied to obtain multiferroic composites. The DC electrical conductivity, microstructure, crystal structure, and magnetic, dielectric, and ferroelectric properties of the multiferroic composites were investigated. Research has shown that incorporating two ferroelectric materials into the composite improves performance parameters, thereby preserving the high magnetic and dielectric properties of the composite materials. The BT-BF-F exhibits better magnetic properties but weaker ferroelectric parameters and higher dielectric loss. In contrast, the BT-PZT-F shows high ferroelectric properties with appropriate saturation of the *P-E* hysteresis loop, but weaker magnetic properties. Temperature-dependent dielectric studies of the composite materials revealed the effects of phase transitions between the composite components and the influence of magnetic ferrite on the dielectric properties. Multiferroic ceramic composites based on magnetic and double-ferroelectric materials exhibit interesting functional properties suitable for applications in modern microelectronics.

## 1. Introduction

Ceramic ferroelectric materials exhibiting spontaneous polarization belong to the group of materials with extended properties (dielectric, piezoelectric, and pyroelectric), which have found versatile applications in many fields of microelectronics, micromechanics, and micromechatronics [1,2,3,4,5,6]. Ferroelectrics are used, among other applications, as resonators, filters, ultrasonic transducers (medical ultrasound devices, non-destructive testing (NDT), and flow sensors), infrared detectors (thermal imaging cameras and motion sensors), pressure, vibration, and acceleration sensors, light modulators, piezoelectric igniters, and precision micropositioners (in AFM microscopes) [7,8,9]. Permanent polarization in ferroelectric materials has also been used for fast data recording in FRAM memories [10,11]. The best-known ferroelectric materials with the widest application potential include barium titanate BaTiO_3_ (BT) [12,13,14,15,16,17], doped solid solutions based on PbZr_1-_*_x_*Ti*_x_*O_3_ (PZT) [18,19,20,21,22], solid solutions based on (1-*x*)PbMg_1/3_Nb_2/3_O_3_-*x*PbTiO_3_ [23,24], PbFe_1/2_Nb_1/2_O_3_ [25,26,27], and bismuth ferrate BiFeO_3_ [28,29,30,31,32]. The magnetic properties of these materials are widely used in many fields of advanced technology [33,34,35,36]. Ferrite materials are categorized into four types, i.e., spinel, garnet, hexagonal, and orthoferrites, based on their crystal structures and magnetic properties, and the most important of these are spinel-structured ferrites [37]. Soft magnetic materials are used, among others, as electromagnetic interference (EMI) suppressors, inductors, microtransformers, and different types of sensors, and as transformer cores that perform excellently in high-frequency applications [37,38,39]. Permanent magnets are used in industry in the automotive, acoustic, renewable energy technology, home appliances, and electronics sectors [38,40]. Magnetic materials, thanks to their unique properties, have also found special applications in engineering, e.g., magnetic recording media, magnetic seals, and ferrofluids; in medicine, e.g., in magnetotherapy, magnetic separation, magnetic hyperthermia, magnetic resonance imaging, and drug delivery; or in the recycling industry as separators of ferrous and non-ferrous metals [41]. The group of magnetic materials of high interest includes ferrites, including nickel–zinc [42,43,44], cobalt-zinc [25], nickel–cobalt [45], manganese–zinc [46], and nickel–zinc–copper [47]. Additional unique functional properties of modern materials are achieved by combining magnetic and ferroelectric materials in the form of composites, thin layers, or sandwich systems [48,49,50]. The advantage of this type of multiferroic system is the ability to leverage the physical properties of its individual components, which expands the application possibilities of new functional materials to meet the ever-increasing civilizational demand to improve and facilitate human life. Multiferroic materials, which combine at least two ferroic ordering states (ferroelectric, ferromagnetic, or ferroelastic) and their coupling, allow, for example, control of a magnetic subsystem using an electric field (and vice versa) [51,52,53,54,55,56]. Magnetic materials typically have high electrical conductivity, which is a major limitation from their application in multiferroic connections [33]. Among ferrite materials, nickel–zinc ferrites exhibit good magnetic properties while maintaining relatively high resistance at room temperature [33]. For example, the nickel–zinc ferrite Ni_0.64_Zn_0.36_Fe_2_O_4_ is a soft ferrite with magnetic induction *B* = 380 mT and high resistivity (~10^5^ Ωm) at RT, a phase transition at about 350 °C, and a working frequency range of 50–1000 MHz [57,58]. Multiferroic materials are used as microelectromechanical actuators, sensors, magnetoelectric transducers, microwave filters, spintronic devices, energy harvesters, and new-generation multiferroic memories (MRAMs) [9,59,60]. One of the fundamental advantages of using multiferroic materials in microelectronic devices and systems is the potential for significant energy reduction. A key aspect in the widespread application of new materials with functional properties is ensuring repeatable properties in large-scale production, including maintaining the temporal and temperature stability of functional parameters [61]. Therefore, in addition to economic and production considerations, selecting the appropriate technology for producing multiferroic materials is crucial. Many sintering methods are used to achieve optimal functional properties in multiferroics and piezoelectric materials, e.g., spark plasma sintering, microwave sintering, free sintering (pressureless), hot pressing, and cold sintering process [62,63,64,65,66], thereby enabling the production of materials with high functional properties.

Numerous studies on multiferroic ceramic materials indicate that achieving strong ferroelectric–ferromagnetic coupling requires meeting specific conditions, such as high magnetostriction in the ferromagnetic component and high piezoelectric (ferroelectric) parameters in the ferroelectric component, a state of equilibrium between the individual phases, and ideal contact between the ferroelectric and ferromagnetic grains [67]. Practical experience shows that not every combination of constituent materials in a designed multiferroic composite yields the expected results; the presence of a ferrite material tends to degrade ferroelectric and piezoelectric properties while increasing electrical conductivity and dielectric loss. These challenges necessitate finding an appropriate compromise when designing multiferroic composite materials to achieve optimal parameters. For instance, when designing and fabricating multiferroic composites, high ferroelectric properties can be preserved by carefully selecting constituent phases (e.g., ensuring the ferroelectric phase predominates over the magnetic phase) or by employing two ferroelectric materials to create multi-component multiferroic composites.

In this paper, the above modification procedures were proposed to obtain two ternary composite materials (ferroelectric and magnetic) with different ferroelectric components to enhance their ferroelectric and dielectric properties. The technology and functional properties of two ternary multiferroic composites (at a 90/10 ferroelectric/magnetic ratio) were presented using the free sintering method. The composite comprised two ferroelectric components with distinct electrophysical properties, thereby maintaining the high dielectric and magnetic properties of multiferroic materials. The components of the first BT-PZT-F composite were ferroelectric BaTiO_3_ (BT), a ferroelectric PZT solid solution doped with strontium and chromium (PZT), and Ni_0.64_Zn_0.36_Fe_2_O_4_ ferrite (F). The components of the second BT-BF-F composite were ferroelectric BaTiO_3_ (BT), ferro-electro-magnetic BiFeO_3_ (BF), and magnetic zinc–nickel ferrite (F). In the ferroelectric subsystem, the two ferroelectric components were present in equal proportions (50/50). The DC electrical conductivity, microstructure, crystal structure, and magnetic, dielectric, and ferroelectric properties of the multiferroic ceramic composites were investigated.

## 2. Materials and Methods

### 2.1. Materials

Composite materials technology employs a combined process to obtain the test material: high-energy powder grinding, calcination, and pressureless sintering of composite samples. In the experiment, two composite material compositions were designed, each containing three main components: two ferroelectric matrix components (with a total share of 90%) and a magnetic component with a 10% share in the multiferroic composite. The technological process analyzed the effect of grinding time on component synthesis and grain uniformity using a BT-PZT-F composite sample (described in detail in Section 2.1.1). A high-energy planetary mill (Fritsch, Pulverisette-6, Idar-Oberstein, Germany) was used (ZrO_2_ container and balls, rotational speed 200 rpm). After mixing intervals of 1 h, 2 h, 5 h, 10 h, and 20 h, control samples of the mixed powder were collected for X-ray examination (Figure S1, Supplementary Materials). Based on the obtained results, the optimal mixing time for the BT-PZT-F and BT-BF-F composite powders was determined to be 20 h. After high-energy grinding (for 20 h), the composite powders were heated in a muffle furnace for 3 h at 900 °C.

#### 2.1.1. BT-PZT-F Multiferroic Composite

In the BT-PZT-F composite, the ferroelectric component consisted of two materials: BaTiO_3_ (BT) and a strontium- and chromium-doped PZT-type solid solution with the chemical composition Pb_0.94_Sr_0.06_(Zr_0.46_Ti_0.54_)_0.99_Cr_0.01_O_3_ (PZT). The ferroelectric components BT and PZT were weighed in a 50/50 ratio. The technological process used commercial BaTiO_3_ material (99.5%, Sigma-Aldrich, St. Louis, MO, USA), which was additionally calcined at 1350 °C and for 5 h. To obtain the PZT material, classical powder technology was used, in which simple oxides were used: TiO_2_ (99.99%, Sigma-Aldrich, St. Louis, MO, USA), SrCO_3_ (99.99%, Chempur, Piekary Śląskie, Poland), ZrO_2_ (99.5%, Sigma-Aldrich, St. Louis, MO, USA), PbO (99.99%, POCH, Gliwice, Poland), and Cr_2_O_3_ (99.99%, POCH, Gliwice, Poland). The component powders were mixed in a planetary ball mill for 12 h, then synthesized at 850 °C for 2 h. The magnetic component in the composite was zinc–nickel ferrite with the chemical composition Ni_0.64_Zn_0.36_Fe_2_O_4_ (F). Ferrite material was also obtained by classical powder technology using simple oxides: NiO (Sigma-Aldrich, 99.8%, Steinheim, Germany), ZnO (POCH, 99.99%, Gliwice, Poland), and Fe_2_O_3_ (POCH, 99.99%, Gliwice, Poland). The magnetic powders, weighed in stoichiometric proportions, were mixed in a planetary ball mill for 24 h, then sintered at 1000 °C for 4 h. The multiferroic BT-PZT-F composite was obtained by combining a 90% ferroelectric component (i.e., two ferroelectric materials, BT and PZT, in a 50/50 ratio) with a 10% magnetic component. The individual composite components were weighed in the proper amount and mixed in a planetary ball mill for 20 h. The powders were then formed into disk-shaped compacts and pressed on a hydraulic press. Final sintering of the multiferroic composite samples was achieved by pressureless sintering at 1250 °C for 2 h.

#### 2.1.2. BT-BF-F Multiferroic Composite

In the BT-BF-F composite, the ferroelectric component consisted of two materials: BaTiO_3_ (BT) and BiFeO_3_ (BF) weighed in a 50/50 ratio, and the magnetic component was, as before, zinc–nickel ferrite with the chemical composition Ni_0.64_Zn_0.36_Fe_2_O_4_ (F). In the technological process, the BaTiO_3_ material was prepared as described in Section 2.1.1, while BiFeO_3_ was obtained by classical powder technology using the simple oxides Fe_2_O_3_ (>99.98%, Sigma-Aldrich, St. Louis, MO, USA) and Bi_2_O_3_ (99.9%, Sigma-Aldrich, St. Louis, MO, USA). The component powders were mixed in a planetary ball mill for 12 h, and then the synthesis was carried out at 700 °C for 4 h. The magnetic component of the composite, i.e., Ni_0.64_Zn_0.36_Fe_2_O_4_, was prepared as described in Section 2.1.1. The multiferroic BT-BF-F composite was obtained from a combination of a ferroelectric component (i.e., two ferroelectric compositions, BT and BF, in a 50/50 ratio) in the amount of 90% and ferrite (F) in the amount of 10%. After appropriate weighing, the powders were mixed in a planetary ball mill for 20 h, then formed into disk-shaped compacts and pressed on a hydraulic press. Sintering of the multiferroic composite samples was performed by pressureless sintering (i.e., free sintering method) at 1250 °C for 2 h.

### 2.2. Characterizations

After the sintering process, the multiferroic composite samples were subjected to mechanical processing (grinding) and then annealed at 600 °C for 15 min to remove mechanical stresses. For electrical measurements, silver electrodes were applied to both surfaces of the composite sample. The microstructure and EDS (Energy Dispersive Spectrometry) of the multiferroic composites were carried out on the Field Emission Scanning Electron Microscopy JSM-7100F TTL LV (Jeol Ltd., Tokyo, Japan). The microstructure images were captured using two techniques, i.e., the SB standard technique (all electrons, both backscattered and secondary electrons, are detected) and the BSE technique (using the signal from the backscattered electron detector). For imaging, the composite samples were coated with gold (Smart Coater DII-29030SCTR, Jeol Ltd., Tokyo, Japan). The X-ray tests of the multiferroic composite powders were performed at room temperature, RT using a Philips PW 1830 (Philips, Almelo, The Netherlands) diffractometer using Cu Kα radiation (*λ* = 0.15418 nm). The magnetic properties were carried out on a SQUID magnetometer, MPMS XL-7 (Quantum Design, San Diego, CA, USA), in the range from −263 °C to 30 °C, and the magnetic field of 0.1 T. Archimedes’ method was utilized to calculate the relative density of the multiferroic composites. Dielectric tests were performed using the QuadTech 1920 Precision LCR Meter (QuadTech, Maynard, MA, USA) in a frequency range of 20 Hz to 1 MHz and the temperature range from RT to 500 °C (800 °C). In DC electrical conductivity measurements taken over high- and low-resistance ranges using the Keysight 34465A (Keysight Technologies, Inc., Santa Rosa, CA, USA) digital multimeter and the Keithley 6517B (Keithley, Cleveland, OH, USA) electrometer/high-resistance meter. *P-E* hysteresis loops were performed at RT and at 5 Hz using a virtual Sawyer-Tower circuit, supported by a high-voltage amplifier (Matsusada Inc., HEOPS-5B6 Precision, Kusatsu, Japan), and a digital card (NI USB-6002, National Instruments Corporation, Austin, TX, USA). A General Electric Phoenix v|tome|x (GE Sensing & Inspection Technologies GmbH, Wunstorf, Germany) micro-computed tomography scanner was used to estimate internal porosity of composite samples (scanning of internal microstructure cross-sections). The captured microstructure images were processed in Volume Graphics^®^ VGSTUDIO MAX (Volume Graphics GmbH, Heidelberg, Germany), and visualization, animation, and detailed analysis were performed using the Volume Graphics^®^ myVGL viewer (Volume Graphics GmbH, Heidelberg, Germany). Porosity analysis was performed on the microstructure photos using ImageJ software (ImageJ 1.37v, LOCI, University of Wisconsin-Madison, WI, USA).

## 3. Results

### 3.1. X-Ray Study

**Figure 1:**
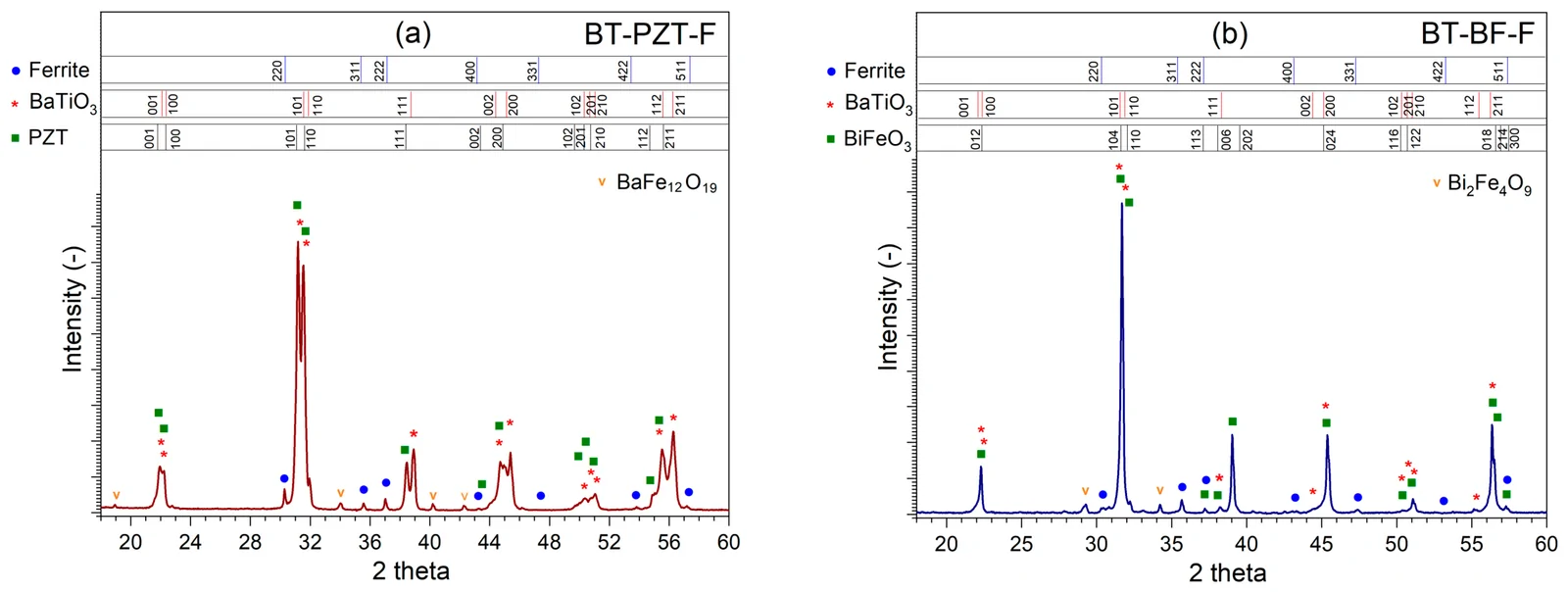
X-ray diffraction patterns of BT-PZT-F (**a**) and BT-BF-F (**b**) composite materials.

Figure 1 presents the results of X-ray examinations for composite samples. In the case of the BT-PZT-F composite sample (Figure 1a), strong reflections were recorded for two ferroelectric materials: BaTiO_3_ (BT) and Pb_0.94_Sr_0.06_(Zr_0.46_Ti_0.54_)_0.99_Cr_0.01_O_3_ (PZT), which constituted 90% of the composite matrix. In the case of BT, the best fit to the experimental results was obtained using the ICDD 00-005-0626 pattern (tetragonal phase with space group *P*4*mm*). In the case of the PZT-type material, X-ray examination revealed the presence of tetragonal (T) phase, near the morphotropic region (T + Re). The best fit was obtained for the JCPDS card no. 04-015-7391 (space group *P*4*mm*). In the X-ray pattern, weak peaks originating from ferrite Ni_0.64_Zn_0.36_Fe_2_O_4_ (F) were matched to the pattern card no. 01-077-9718 (spinel structure, space group *Fd*-3*m*). X-ray analysis also revealed trace amounts of a foreign phase (BaFe_12_O_19_). In the case of the BT-BF-F composite sample, strong reflections were recorded for two ferroelectric materials, BaTiO_3_ (ICDD 00-005-0626 pattern) and BiFeO_3_ (BF), as well as weak peaks originating from ferrite Ni_0.64_Zn_0.36_Fe_2_O_4_ (01-077-9718 pattern). The best fit to the experimental results for the BF material was obtained using the ICDD 00-066-0439 pattern (a rhombohedral structure belonging to space group *R*3*c*). X-ray analysis also revealed trace amounts of a foreign phase (Bi_2_Fe_4_O_9_).

### 3.2. FESEM Microstructure and EDS Analysis

**Figure 2:**
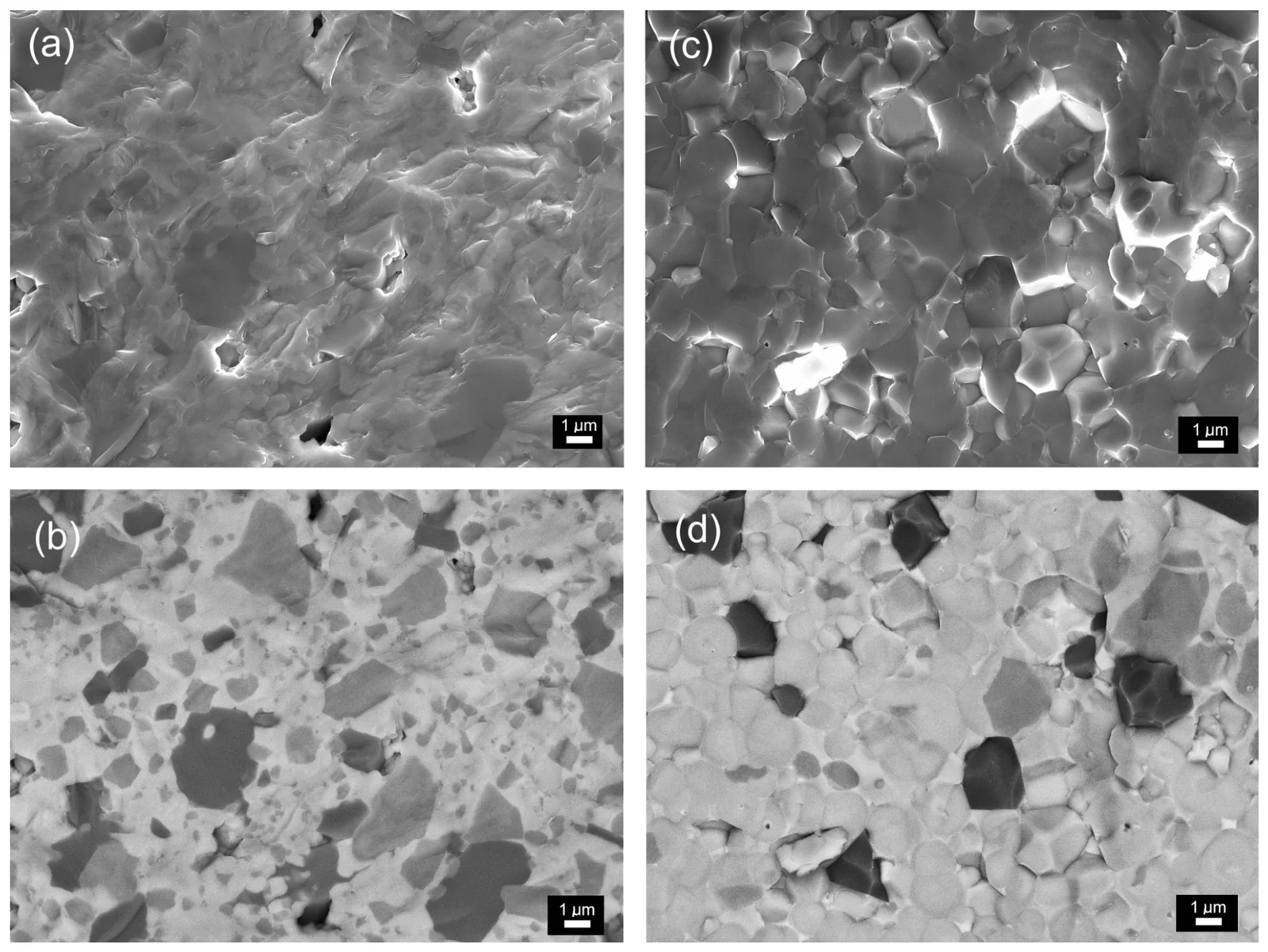
Cross-section FESEM images of BT-PZT-F (**a**,**b**) and BT-BF-F (**c**,**d**) multiferroic composites made by SB technique (**a**,**c**) and BSE technique (**b**,**d**).

**Figure 3:**
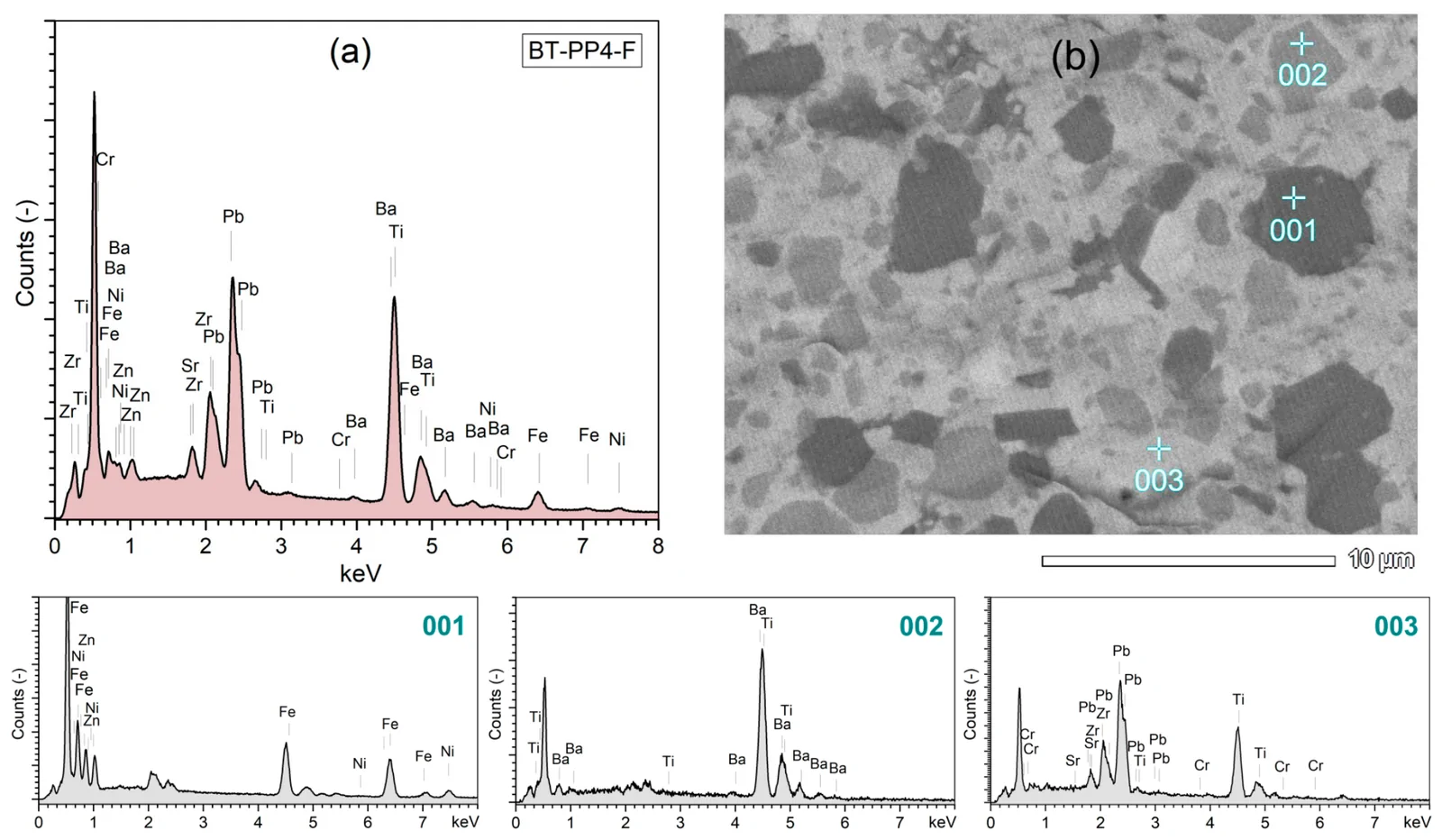
Surface EDS analysis of BT-PZT-F multiferroic composite (**a**), BSE-FESEM microstructure images with point EDS analysis (**b**); F (001), BT (002), and PZT (003).

**Figure 4:**
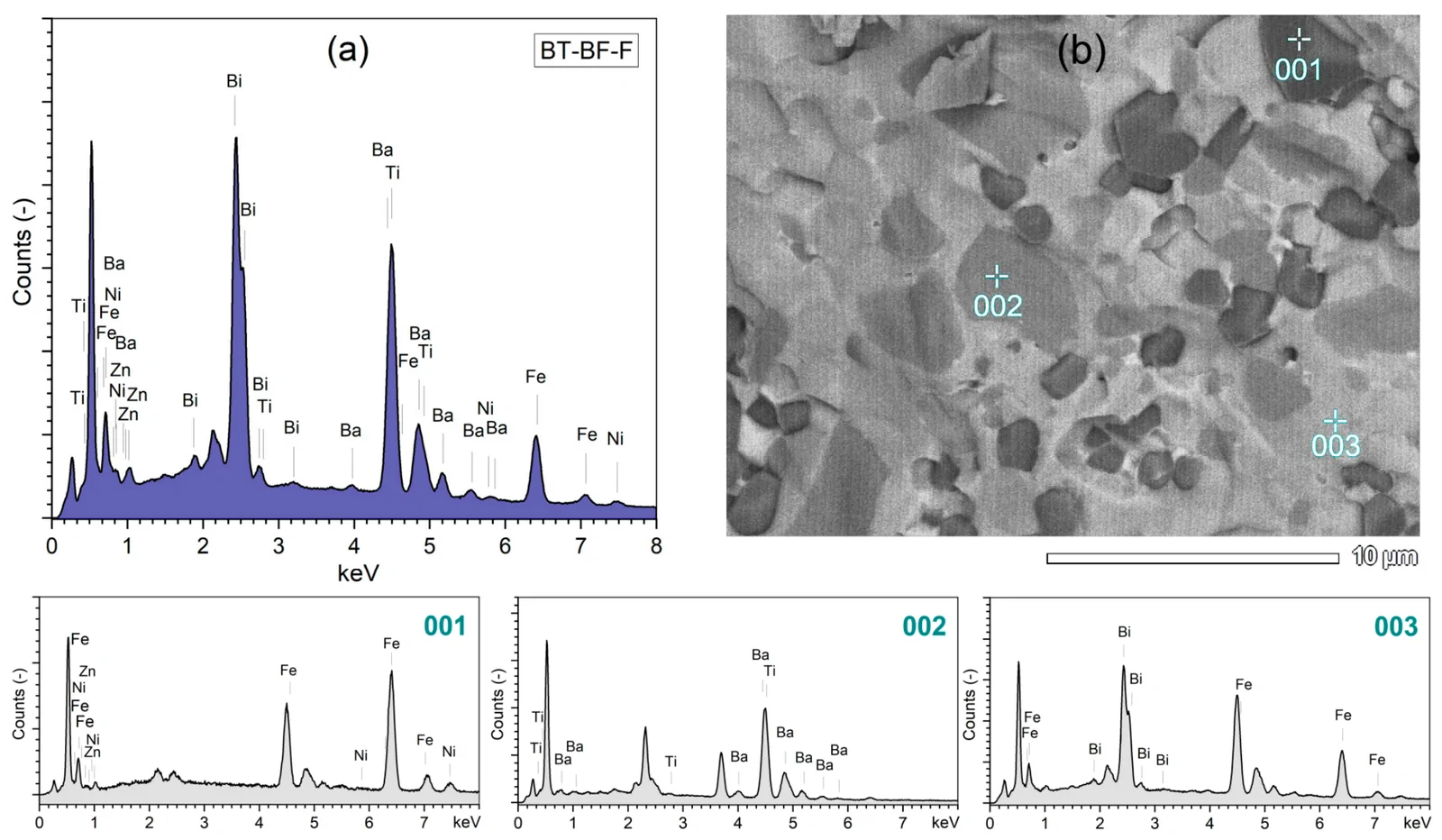
Surface EDS analysis of BT-BF-F multiferroic composite (**a**), BSE-FESEM microstructure images with point EDS analysis (**b**); F (001), BT (002), and BF (003).

Two types of imaging capture were used in the FESEM measurements: the SB technique (i.e., combined signals from secondary and backscattered electrons) and the BSE technique (i.e., signals from the backscattered electron detector). BSE observation provides visualization of the microstructure of multiferroic composites, revealing two types of ferroelectric regions and magnetic regions (Figure 2). This is possible because the presence of elements with higher atomic numbers (components of the ferroelectric composite matrix) is revealed by the BSE detector as bright regions. On the other hand, elements with lower atomic numbers (i.e., the magnetic component of the composite) are revealed as dark regions. Therefore, BSE microstructure imaging is particularly useful for observing the microstructure of multiferroic ceramic materials with magnetic and ferroelectric grains, which are composed of elements with different atomic masses (in the analyzed composites, the difference in atomic masses between magnetic and ferroelectric grains is significant). This allowed us to visualize and distinguish three types of grains with different shades in the microstructure of multiferroic composites, i.e., light grains represented by the “heavier” component of the ferroelectric matrix, namely PZT (for BT-PZT-F) and BF (for BT-BF-F) composites, and gray grains represented by the “lighter” component of the ferroelectric matrix, i.e., BT, whereas dark grains represented by the magnetic ferrite, F. The identification of grains of individual composite components was confirmed by point EDS analysis (Figure 3 and Figure 4). Figure 2 presents FESEM microstructural images of multiferroic composites obtained using the above imaging techniques (i.e., SB and BSE), highlighting the advantage of the BSE technique in studying the microstructure of multiferroic composite materials. In the BT-PZT-F composite sample, the microstructure is characterized by pronounced solidification and intense sintering of the ceramic grains (Figure 2a). Such strong sintering results in a lack of visible grain boundaries in the sample cross-section, which, in standard SB imaging, prevents visualization of the distribution of ferroelectric and magnetic components. The BSE technique (Figure 2b) clearly exposed the microstructure grains belonging to the PZT ferroelectric (light grains), the BT ferroelectric (gray grains), and the magnetic grains belonging to ferrite (dark grains). FESEM microstructural examination of the BT-PZT-F composite showed that the two components of the composite matrix are randomly distributed in the sample volume, similar to the stochastic distribution of magnetic ferrite grains in the composite matrix. Moreover, in the microstructure of the BT-PZT-F composite sample, inhomogeneity of grain sizes of individual composite components is observed.

In the case of the BT-BF-F composite sample (Figure 2c), grain boundaries in the microstructure are more clearly visible than in the case of the BT-PZT-F composite. The microstructure is characterized by low porosity and strong sintering of ceramic grains. A visualization of the grain distribution of individual composite components in the microstructure is shown in Figure 2d using the BSE technique. Grains of the ferroelectric matrix, i.e., the “heavier” BF material, are exposed as light grains, while those of the “lighter” BT material are exposed as gray grains. Grains of the magnetic component (ferrite) are randomly distributed within the ferroelectric matrix and appear as dark grains. In the microstructure of the BT-BF-F composite sample, the grain size uniformity of individual composite components is greater compared to the BT-PZT-F composite sample. The results of surface (a) and point (b) EDS analyses of multiferroic composite samples are shown in Figure 3 for BT-PZT-F and in Figure 4 for BT-BF-F. Surface EDS analysis of a larger surface area of the composite materials revealed the presence of all the composite constituent elements and the absence of foreign elements and impurities. The percentages of the composite elements in the samples are summarized in Table 1. Figure 3b and Figure 4b present FESEM images obtained by the BSE method along with point EDS analysis of the fracture microstructure of the composite samples. In the case of the BT-PZT-F sample, the EDS point analysis (Figure 3b) confirmed that in the dark areas of the microstructure, the elements of the magnetic component of ferrite Ni_0.64_Zn_0.36_Fe_2_O_4_ (analysis 001) dominate, i.e., Zn, Ni, and Fe; in the gray areas, there are elements of the ferroelectric component BaTiO_3_ (analysis 002), i.e., Ba and Ti. In contrast, in the light areas there are constituent elements of the second ferroelectric material PZT, i.e., Pb_0.94_Sr_0.06_(Zr_0.46_Ti_0.54_)_0.99_Cr_0.01_O_3_ (analysis 003), namely Pb, Zr, Ti, Sr, and Cr.

In the case of the BT-BF-F multiferroic composite sample, the EDS point analysis (Figure 4b) confirmed that in the dark areas of the microstructure there are elements of the magnetic ferrite Ni_0.64_Zn_0.36_Fe_2_O_4_ (analysis 001), i.e., Zn, Ni, and Fe, in the gray areas the elements of the ferroelectric component BaTiO_3_ (analysis 002), i.e., Ba and Ti, dominate, whereas in the light areas there are constituent elements of the second ferroelectric component BiFeO_3_ (analysis 003), i.e., Bi and Fe.

### 3.3. Internal Porosity Testing Using Microtomography

**Figure 5:**
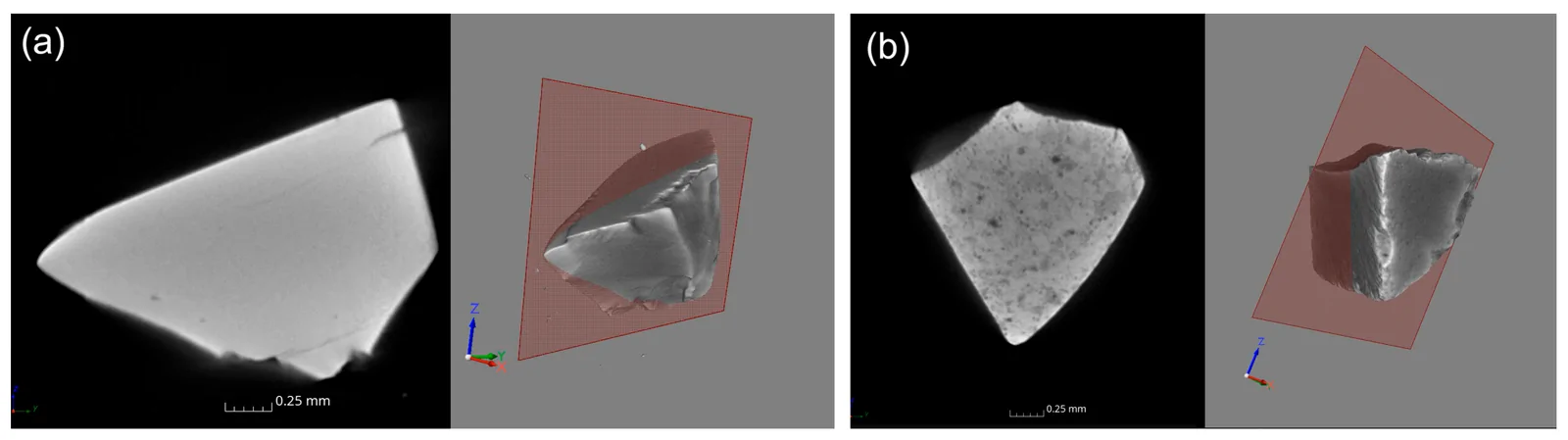
Microstructure images of multiferroic composites BT-PZT-F (**a**) and BT-BF-F (**b**) taken in a random thickness scanning section in a microtomographic study.

**Figure 6:**
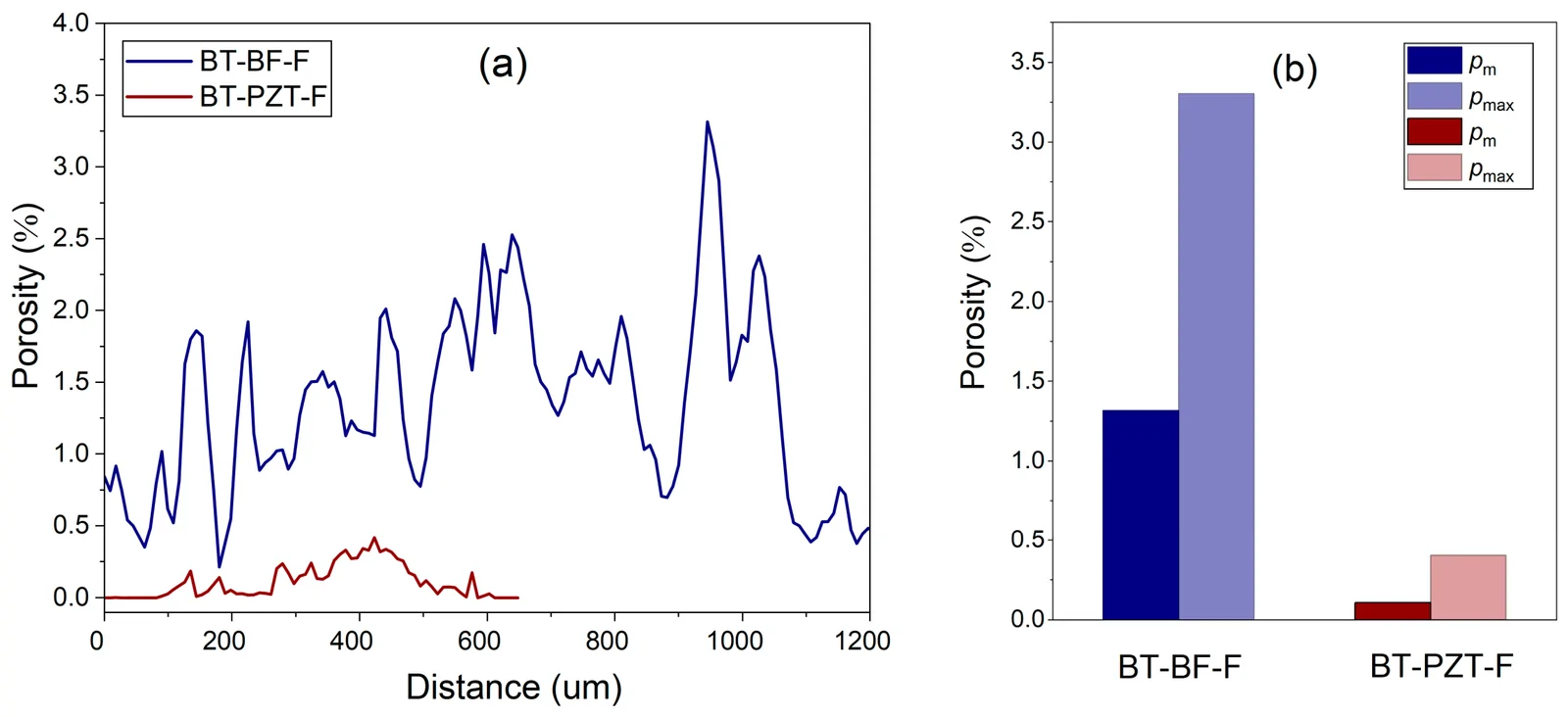
Distribution of internal porosity in the microstructure of composite samples obtained from thickness scanning (**a**) and the average porosity value and the maximum recorded porosity (**b**).

A representative image of the internal microstructure of multiferroic ceramic composites, obtained at a random location during the microtomographic examination, is shown in Figure 5. A complete visualization of the internal microstructure of the BT-PZT-F (a) and BT-BF-F (b) composite samples obtained from the microtomographic examination (in the form of an animation) is included in the Supplementary Materials as *wmv* files (Video S1, Supplementary Materials ). The analysis is a compilation of scanned sections of the microstructures within the composite samples. Based on the microtomographic images obtained by scanning through the thickness of the samples, the internal porosity of the composite materials was estimated. Figure 6 presents the results of the internal porosity analysis of composite materials obtained by analyzing successive microstructural sections captured across the samples’ thickness.

The BT-PZT-F composite exhibits greater microstructural uniformity throughout the volume and low internal porosity. The average internal porosity *p*_a_ revealed in successive thickness scan sections (for a 0.65 mm sample thickness) is 0.107%, whereas the maximum porosity *p*_max_ revealed in the microstructure of the scanned sample volume is 0.405%. For the BT-BF-F composite, scanning was performed over a larger measurement section due to the thicker sample (1.2 mm). Microtomographic analysis revealed significantly higher internal porosity, with areas of the microstructure where it exceeded 2%. In the BT-BF-F composite, the average internal porosity *p*_a_ revealed by thickness scanning of the sample is 1.317%. In contrast, the maximum porosity *p*_max_ revealed in the microstructure of the scanned sample volume is 3.304% (at a thickness of 0.95 mm of the sample in Figure 6a).

### 3.4. Dielectric Properties

**Figure 7:**
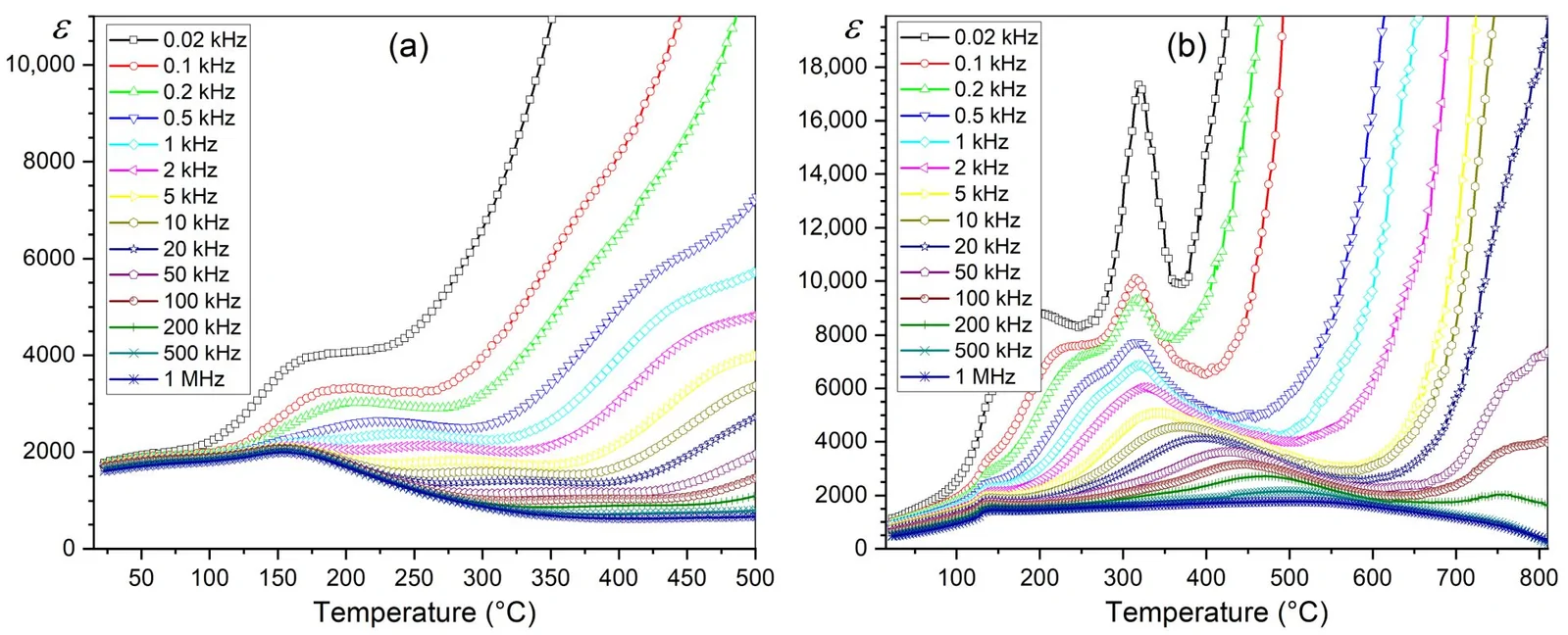
Temperature dependence of permittivity for BT-PZT-F (**a**) and BT-BF-F (**b**) multiferroic composites.

Previous experiments and studies [43] have shown that multiferroic composite materials obtained by connecting a ferroelectric material with a ferrite usually exhibit deterioration in ferroelectric and dielectric properties, but also an increase in electrical conductivity and dielectric loss. In temperature-dependent dielectric tests of ternary composite materials, phase transitions in the two ferroelectric materials interpenetrate, creating broad, local phase-transition blurs. For BT, the phase transition typically occurs at approximately 130 °C [68], while for PZT, it occurs at approximately 355 °C [69]. In the case of the BT-PZT-F sample, dielectric tests were performed over the range from RT to 500 °C (Figure 7a), and permittivity, *ε*, at RT at 1 kHz is 1094. With increasing temperature, there is a systematic increase in permittivity, with higher values observed at low frequencies that decrease with increasing frequency. Combining two ferroelectric materials in equal amounts (50/50) causes the superposition of their ferroelectric–paraelectric phase transitions, which results in interpenetration and their broad blurring. Temperature-dependent plots of permittivity exhibit a complex nature, revealing regions characterized by different types of dielectric dispersion. At high temperatures and low frequencies, electrical conduction effects dominate, manifesting as a relatively rapid rise in the permittivity curves [70]. On the other hand, the frequency increases (100 kHz–500 kHz), this type of dielectric dispersion shifts toward higher temperatures, where a new type of dispersion—marked by the appearance of a local maximum—begins to dominate. This type of dispersion is observed primarily in relaxor materials or dipole-glass-like compounds [70]. In multiphase materials at higher temperatures, dispersion also arises from inhomogeneities at phase boundaries. Such irregularities can generate additional free charge carriers at the interfaces and induce local changes in polarization [71]. This phenomenon is known as interfacial polarization (or Maxwell–Wagner polarization) [70] and can constitute a dominant component of the total conductivity in multiferroic composite materials [72,73]. The complex nature of the temperature dependence *ε*(*T*) of the BT-PZT-F composite—resulting from the phenomena described above—may also be influenced by an additional factor: the presence of magnetic ferrite in the composite composition. A magnetic phase transition of the ferrite occurs at 350 °C; this may serve as an additional factor contributing to the intensification of the observed high-temperature dielectric dispersion (Figure 7a).

In the case of the BT-BF-F sample, dielectric tests (Figure 7b) were conducted in a wider temperature range, i.e., from RT to 810 °C, due to the very high phase transition temperature of the ferroelectric BF (about 825 °C) [74]. At RT and 1 kHz, the permittivity is 882. For the BT-BF-F composite, the plot of permittivity versus temperature shows behavior similar in complexity to that of the BT-PZT-F composite sample, but with even greater intensity at elevated temperatures. This may be attributed to the heightened influence of the factors described above in this composite material, as well as the stronger impact of the ferrite phase transition on its dielectric properties. In turn, the rapid increase in permittivity, starting already at 450 °C for 20 Hz, is related to the initiation of a broad ferroelectric phase transition in BF, which for 1 kHz occurs at a temperature of 825 °C [74] and is outside the temperature range of the measurement capabilities of the station.

**Figure 8:**
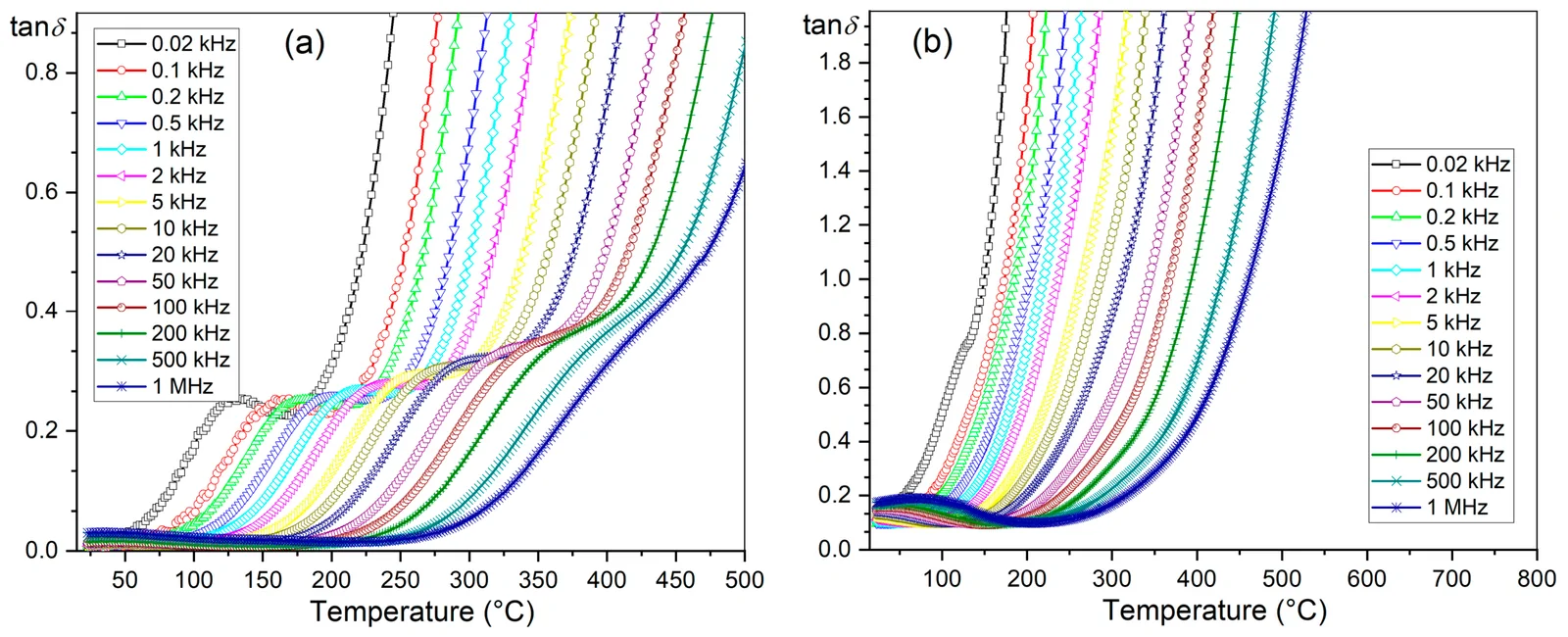
Temperature dependence of dielectric loss for BT-PZT-F (**a**) and BT-BF-F (**b**) multiferroic composites.

The changes in dielectric loss of composite materials are presented in Figure 8, expressed as the dielectric loss tangent (tan*δ*). With increasing frequency, the dielectric loss tangent values of composite materials decrease. At RT and 1 kHz, the tanδ for BT-PZT-F is 0.0106. At higher temperatures, the increase in dielectric loss is significant. For the BT-BF-F composite, at room temperature and 1 kHz, the dielectric loss tangent is 0.1044. The BT-BF-F composite is characterized by higher tan*δ* values and a greater increase in dielectric loss with increasing temperature than is the case for the BT-PZT-F composite. This is related to the more rapid increase in the electrical conductivity of the BT-BF-F sample at higher temperatures, as confirmed by DC electrical conductivity studies presented in Section 3.5.

### 3.5. DC Conductivity

**Figure 9:**
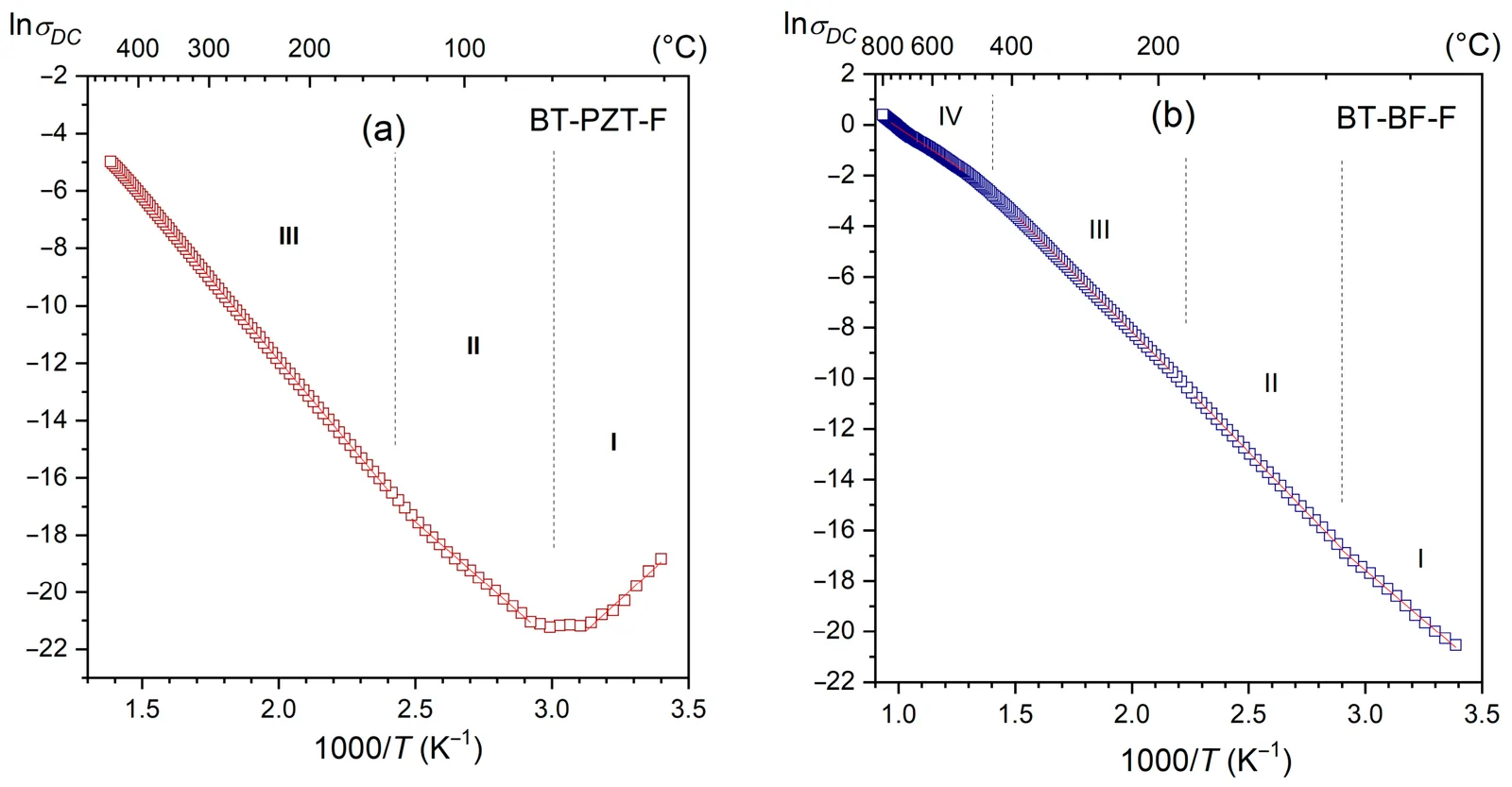
Ln*σ*_DC_(1000/*T*) plot for BT-PZT-F (**a**) and BT-BF-F (**b**) multiferroic composites.

The multiferroic BT-BF-F composite exhibits higher DC electrical conductivity than the BT-PZT-F composite (Figure 9). This is due to the significantly lower resistivity of the second ferroelectric component of the composite, i.e., the BF material, which causes an increase in the electrical conductivity of the entire multiferroic composite. At room temperature, the resistivity *ρ*_DC_ values of both composite materials are similar and amount to 4.6 × 10^8^ Ω⋅m for BT-BF-F and 2.8 × 10^8^ Ω⋅m for BT-PZT-F. In the case of the BT-PZT-F composite, with increasing temperature, there is an initial small decrease in the *σ*_DC_ conductance value (up to approximately 60 °C), followed by a progressive increase in DC electrical conductivity. For the BT-BF-F composite material, the increase in *σ*_DC_ conductance with temperature starts at RT. In contrast, the increase in DC electrical conductivity is much greater than that for BT-PZT-F. On the obtained temperature courses, straight-line sections of the courses were determined (in different temperature ranges), for which activation energies, *E*_a_, were determined according to the Arrhenius equation [75]. In the case of the BT-PZT-F composite, the calculated values of activation energy *E*_a_ are 0.76, 0.72, and 0.98 eV for temperature ranges I, II, and III, respectively. Slightly lower values of activation energy *E*_a_ were recorded for the BT-BF-F composition, which are 0.68, 0.82, 0.79, and 0.53 eV for temperature ranges I, II, III, and IV, respectively. In general, DC conduction in ferrite materials is primarily associated with the hopping of charge carriers between iron ions in different oxidation states (the Verwey hopping mechanism). In contrast, in ferroelectric materials, conductivity depends on the crystal structure and on factors such as the presence of oxygen vacancies and dipole effects associated with defects [75], and, in the case of BT-BT-F composite materials, additionally on bismuth vacancies. In ferroelectric materials, the different activation energy values observed within the specified temperature ranges result from differences in the conduction mechanism and domain configuration [76]. It can be described by the fact that domain wall motion in the tetragonal phase is hindered by the complex domain structure, in contrast to the more readily configurable structure of the rhombohedral phase [77]. In the rhombohedral system, an *E*_a_ of approximately 0.59 eV can be attributed to small polarons formed through the interaction of electrons and/or holes with phonons. In contrast, the *E*_a_ is higher in the tetragonal system. Higher activation energy values (above 0.81 eV) may be associated with oxygen vacancies and the phenomenon accompanying it that arises at elevated temperatures [76]. An increase in temperature promotes domain wall motion; however, the accumulation of excess oxygen vacancies at domain boundaries leads to domain pinning [77]. At higher temperatures, the activation energy *E*_a_ values for the multiferroic composites range from 0.72 to 0.98 eV, which may be related to the presence of oxygen vacancies.

### 3.6. Ferroelectric Properties

**Figure 10:**
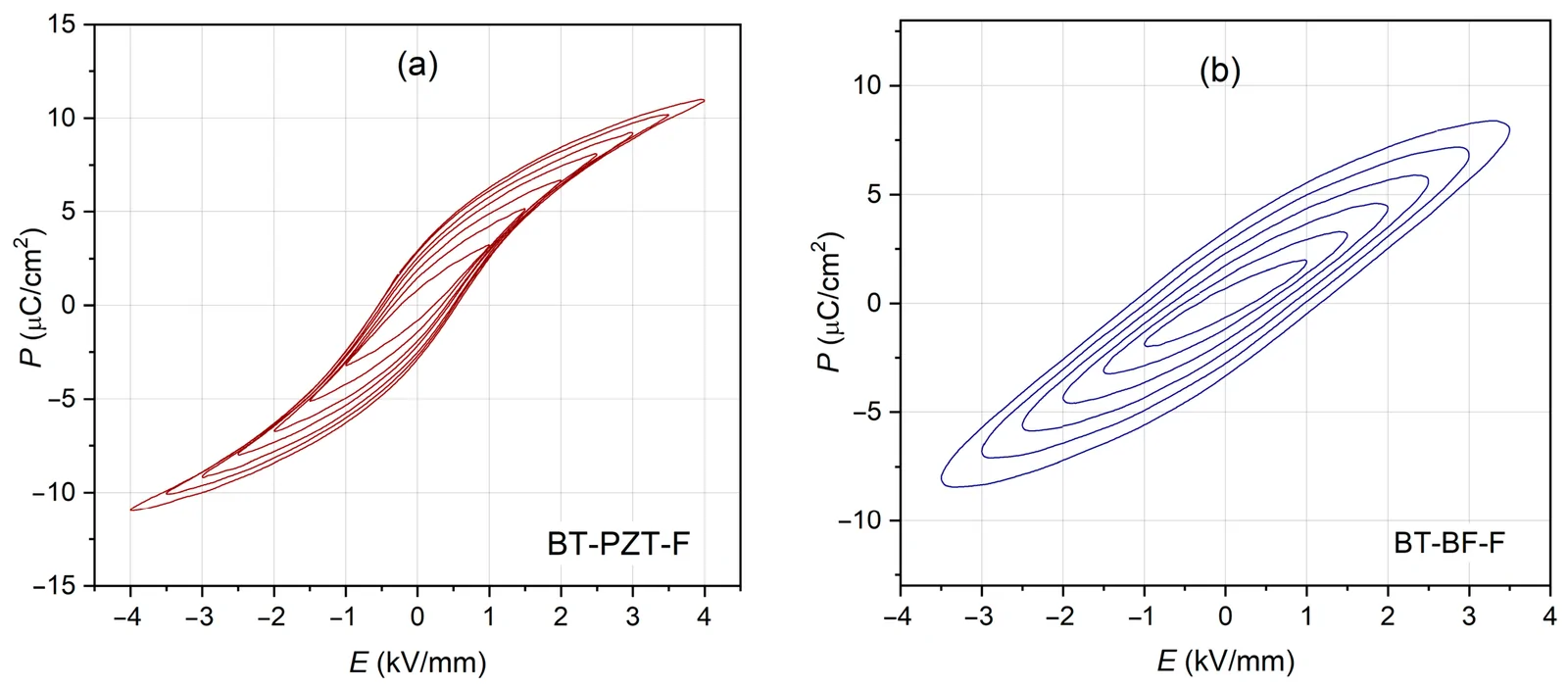
*P–E* hysteresis loops for multiferroic composites; BT-PZT-F (**a**) and BT-BF-F (**b**) (5 Hz, RT).

Figure 10 presents studies of the ferroelectric properties of composite materials, i.e., the *P-E* relationship at room temperature. The graphs compare how the shape of the hysteresis loop changes as the electric field applied to the sample increases. In the case of the BT-PZT-F composite, the *P-E* hysteresis loop is characteristic of soft ferroelectric materials, i.e., symmetrical, and saturates correctly with increasing external field. At 4 kV/mm, the hysteresis loop is strongly saturated with a maximum polarization value of *P*_m_ = 11 µC/cm^2^, remanent polarization value of *P*_r_ = 2.94 µC/cm^2^, and coercive field value of *E*_c_ = 0.54 kV/mm. The high values of ferroelectric parameters of the BT-PZT-F sample are the result of the presence of two materials (BT and PZT) with strong ferroelectric properties, which are not weakened by the presence of ferrite in the composite composition.

**Figure 11:**
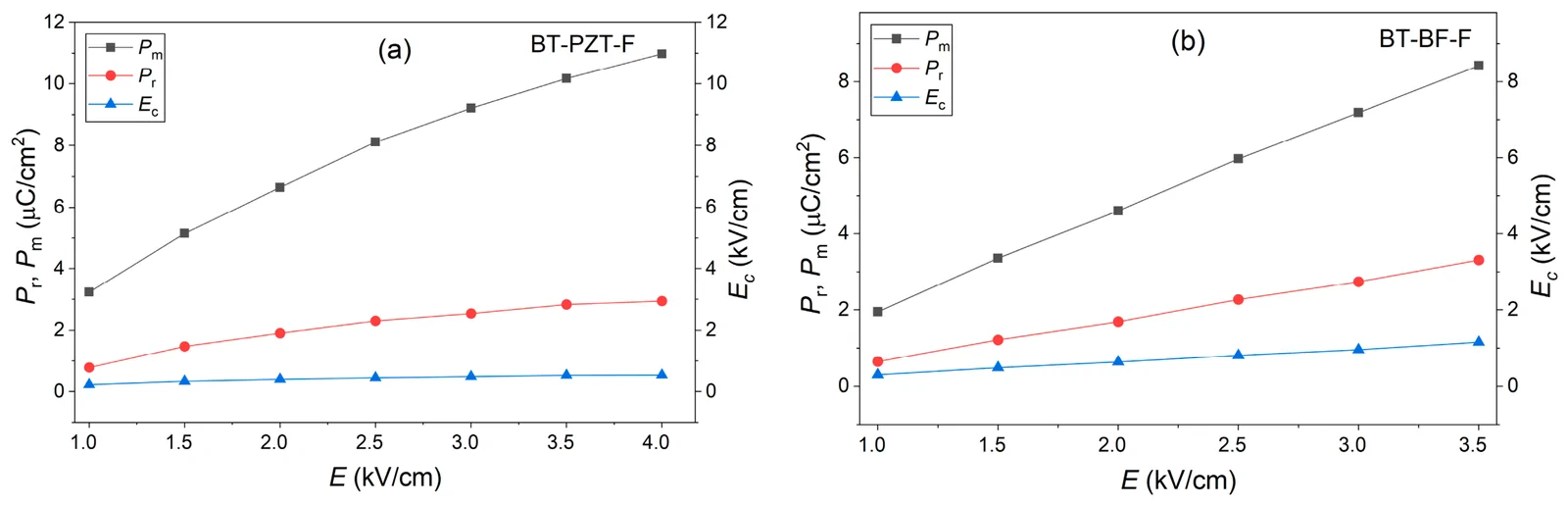
Evaluation of changes in ferroelectric parameters *P*_m_, *P*_r_, and *E*_c_ with increasing electric field of BT-PZT-F (**a**) and BT-BF-F (**b**) multiferroic composites.

The *P-E* hysteresis loop exhibits a different character for the BT-BF-F composite material containing a ferroelectric BT, a magnetic F, and a material with both ferroelectric and magnetic properties, BF. The BT-BF-F ferroelectric hysteresis loop is typical for lossy ferroelectric materials and does not saturate with increasing applied external field, even at 3.5 kV/mm. The higher DC electrical conductivity of the BT-BF-F sample prevents the application of higher electric fields. This is due to the increased share of the magnetic subsystem in the composite (with lower resistance), at the expense of the ferroelectric subsystem. At an applied field of 3.5 kV/mm, the remanent polarization is *P*_r_ = 3.31 µC/cm^2^, and the coercive field is *E*_c_ = 1.16 kV/mm. Figure 11a,b summarize the evaluation of changes in the ferroelectric parameters *P*_m_, *P*_r_, and *E*_c_ as a function of the electric field *E*. For the two composite compositions presented, all ferroelectric parameters increase as the external electric field *E* increases.

### 3.7. Magnetic Properties

**Figure 12:**
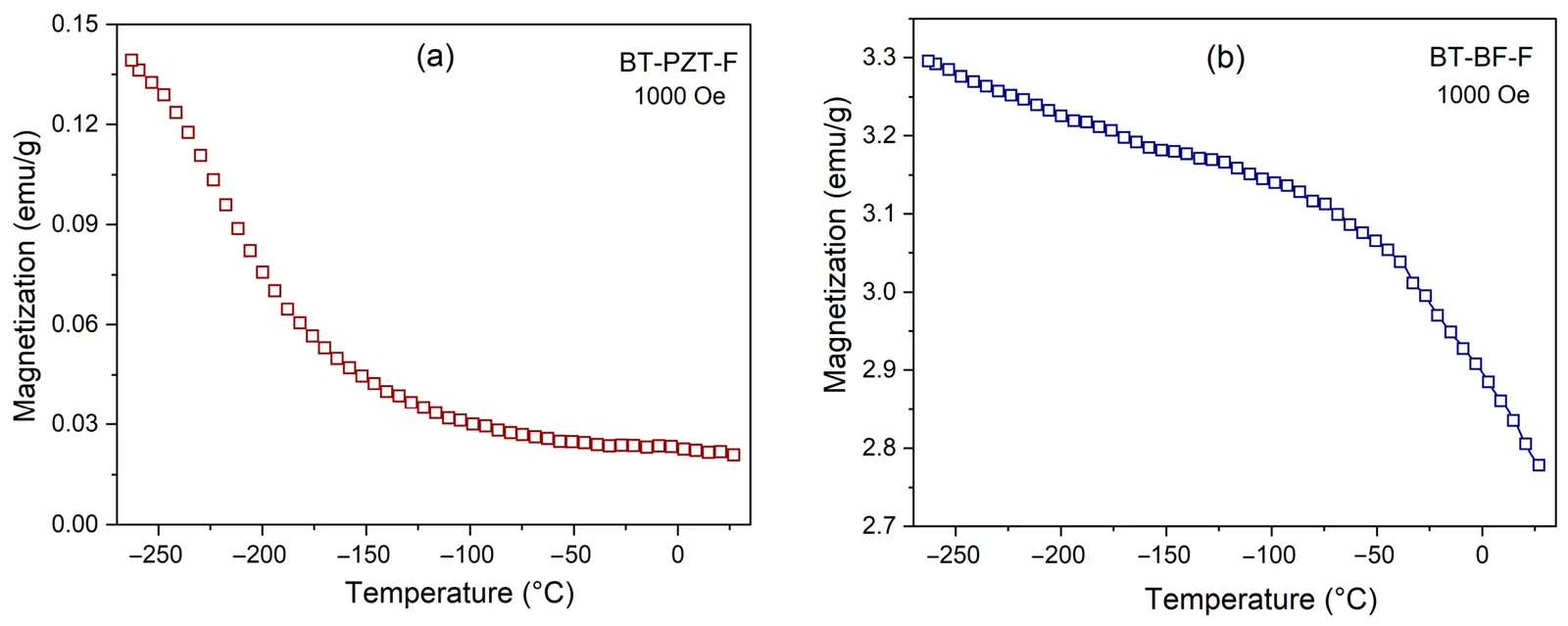
Temperature dependencies of *M* magnetization for BT-PZT-F (**a**) and BT-BF-F (**b**) multiferroic composites.

**Figure 13:**
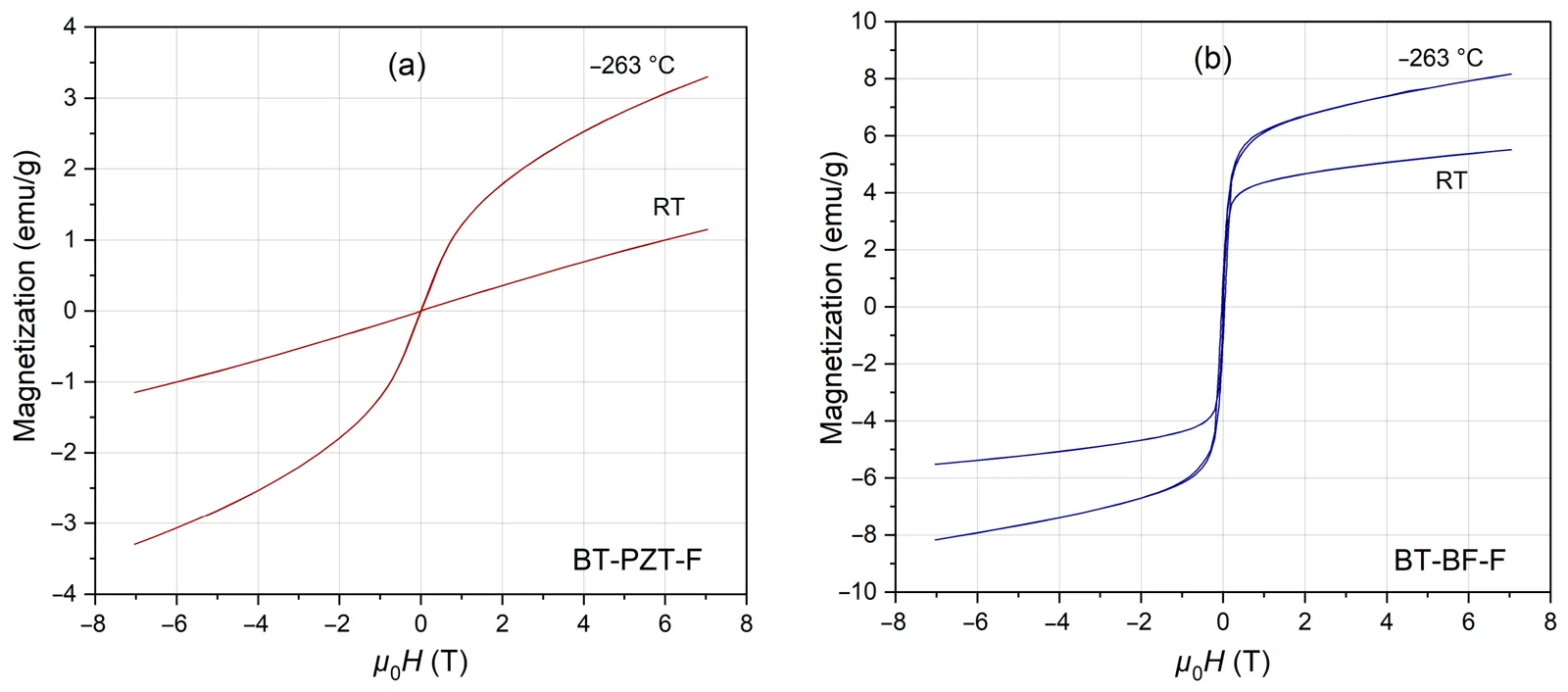
Temperature dependencies of *M-H* magnetic hysteresis loops for BT-PZT-F (**a**) and BT-BF-F (**b**) multiferroic composites, at −263 °C and at RT.

Figure 12 compares the magnetization *M*(*T*) curves for the analyzed composites as a function of temperature. Due to the higher content of iron in the BT-BF-F composite composition, the magnetic response of this composite is significantly greater compared to the BT-PZT-F composite sample. At −265 °C, the magnetization is 3.3 emu/g (for BT-BF-F) and 0.14 emu/g (for BT-PZT-F), while at room temperature, the magnetization values are 2.78 emu/g (for BT-BF-F) and 0.021 emu/g (for BT-PZT-F), respectively. For the BT-BF-F composite sample, the maximum magnetization (*M*) at low temperatures decreases slightly, i.e., to 93% of the initial value (up to −56 °C). Then its decrease is greater, reaching 84% of the initial value at RT. In the case of the BT-PZT-F composite, the *M*(*T*) dependence is different. A sharp decrease in magnetization *M* begins at −263 °C, and at −100 °C, *M* is 0.03, corresponding to 21.43% of the initial value. From −100 °C, the decrease in magnetization is small, and at room temperature is only 15% of the initial value. The magnetic *M-H* hysteresis loops for the compared composite materials also exhibit a different appearance, measured both at −265 °C and RT (Figure 13). In the case of BT-BF-F composite (Figure 13b) with a double ferroelectric material, the magnetic loop *M-H* is slender, highly saturated, and with high maximum magnetization *M*_m_ values (8.20 emu/g for −263 °C and 5.54 emu/g for RT). In contrast, lower magnetic parameters, with lower saturation of the magnetic *M-H* hysteresis loop, occur for the BT-PZT-F sample (Figure 13a). The maximum magnetization *M*_m_ values are 3.30 emu/g at −263 °C and 1.16 emu/g at RT. Slender forms of magnetic *M-H* hysteresis loops are characteristic of soft ferroelectric–ferrimagnetic composite materials exhibiting slim hysteresis loops and low values of the coercive field *H*_c_. The coercive field *H*_c_ value for the multiferroic BT-PZT-F composite is 4.70 × 10^−3^ T at −263 °C and 9.70 10^−4^ T at RT. Higher values of coercive fields are shown by the multiferroic BT-BF-F composite, i.e., 3.60 ×10^−2^ T and 1.80 × 10^−3^ T, for −263 °C and RT, respectively (Table 2).

## 4. Conclusions

A promising approach to obtaining modern engineering materials is to create a variety of physical properties in a single composite material to achieve versatile functionality. The use of two types of ferroelectric compounds in a composite improves performance parameters, thereby preserving the high magnetic and dielectric properties of multiferroic ceramic composites. In this work, two ternary multiferroic composites (BT-PZT-F and BT-BF-F) with ferroelectric (90%) and magnetic (10%) phases were successfully developed and characterized, and their electrophysical properties were investigated. In the first multiferroic composite (BT-PZT-F), the ferroelectric components were BaTiO_3_ (BT) and doped PZT-type material (PZT) (in a 50:50 ratio), whereas the magnetic component was Ni_0.64_Zn_0.36_Fe_2_O_4_ ferrite (F). In the second multiferroic composite (BT-BF-F), the ferroelectric components were BaTiO_3_ (BT) and BiFeO_3_ (BF) materials (in a 50:50 ratio), and the magnetic component was Ni_0.64_Zn_0.36_Fe_2_O_4_ ferrite (F).

X-ray studies revealed strong reflections from the ferroelectric matrix of the composite and weaker ones from the magnetic ferrite. In FESEM studies, BSE microstructure imaging allowed for the visualization and distinguishing of three types of grains with different shades in the microstructure of multiferroic composites, i.e., light grains represented by the “heavier” ferroelectric matrix of PZT (for BT-PZT-F) and BF (for BT-BF-F) composites, gray grains represented by the “lighter” ferroelectric matrix of BT, and dark grains represented by the magnetic ferrite. FESEM analysis revealed a random distribution of magnetic grains within the ferroelectric matrix of the composite samples. Dielectric studies revealed high permittivity values for the multiferroic composites and appropriately low dielectric tangent loss values (maintained up to 150 °C). Temperature-dependent dielectric properties studies revealed the presence of phase transitions in the ferroelectric components of the multiferroic composites, which overlap and interpenetrate. Studies of dielectric properties also revealed a clear influence of the magnetic component on the permittivity of composite materials, whose *ε*(*T*) curves exhibit pronounced dispersion and blurring at high temperatures.

Comparing the obtained composite compositions, it can be concluded that the multiferroic BT-BF-F composite exhibits more favorable magnetic properties, with higher values and greater magnetization stabilization, and saturation of the *M-H* magnetic loop. On the other hand, a higher iron (Fe) content in the composite composition results in a deterioration of the ferroelectric properties (including absence of saturation of the *P-E* ferroelectric hysteresis loop) and an increase in electrical conductivity and dielectric loss. The BT-PZT-F composite exhibits the opposite properties, where the connection of two ferroelectric components, PZT and BF, results in a strong ferroelectric response. This combination provides high ferroelectric properties, strong saturation of the ferroelectric *P-E* hysteresis loop, and high resistance to electrical breakdown in the BT-PZT-F composite sample. On the other hand, a lower iron content in the BT-PZT-F composite reduces the magnetic properties.

The research shows that the appropriate selection of ferroelectric and magnetic components in composite materials enables the desired properties of the final multiferroic material to be obtained for specific applications. Multiferroic composites based on magnetic and double-ferroelectric materials exhibit interesting functional properties suitable for applications in advanced microelectronics.

## Figures and Tables

**Table 1:** Surface EDS analysis for BT-PZT-F and BT-BF-F multiferroic composites.

Elements	BT-PZT-F	BT-BF-F
Ti	13.36	7.55
Cr	0.08	-
Fe	4.18	10.01
Ni	1.27	1.16
Zn	0.76	0.32
Sr	0.93	-
Zr	6.13	-
Ba	25.53	20.86
Pb	28.93	-
Bi	-	43.65
O	18.83	16.45

**Table 2:** Functional parameters of the multiferroic composites.

Parameter	BT-PZT-F	BT-BF-F
*ρ* (g/cm^3^)	6.09	5.91
*p*_a_ (%)	0.107	1.317
*p*_max_ (%)	0.405	3.304
*ε*_r_ at RT ^a^	1094	882
tan*δ* at RT ^a^	0.0106	0.1044
*E*_a_ at I (eV)	0.76	0.68
*E*_a_ at II (eV)	0.72	0.82
*E*_a_ at III (eV)	0.98	0.79
*E*_a_ at IV (eV)	-	0.52
*E*_c_ (kV/mm) ^b^	0.54	1.16
*P*_r_ (μC/cm^2^) ^b^	2.94	3.31
*P*_m_ (μC/cm^2^) ^b^	11	-
*ρ*_DC_ (Ω⋅m) at RT	2.8 × 10^8^	4.6 × 10^8^
*ρ*_DC min_ (Ω⋅m)	1.7 × 10^9^ (at 60 °C)	1.1 × 10^8^ (at RT)
*ρ*_DC_ (Ω⋅m) at *T*_max_	80 (at 450 °C)	0.76 (at 800 °C)
*M* at −263 °C (emu/g)	0.14	3.3
*M* at RT (emu/g)	0.021	2.78
*M*_m_ at −263 °C (emu/g)	3.32	8.20
*M*_m_ at RT (emu/g)	1.15	7.15
*H_c_* (T) at −263 °C	4.7 × 10^−3^	3.6 × 10^−2^
*H_c_* (T) at RT	9.7 × 10^−4^	1.8 × 10^−3^

RT—room temperature, *T*_max_—maximum measuring temperature, ^a^—parametres for 1 kHz, ^b^—parameters for 5 kHz.

## Data Availability

The original contributions presented in this study are included in the article. Further inquiries can be directed to the corresponding authors.

## Supplementary Materials

The following supporting information can be downloaded at: https://www.mdpi.com/article/10.3390/ma19204309/s1, Figure S1: X-ray analysis of BT-PZT-F composite powder after the following mixing times: 1 h, 2 h, 5 h, 10 h, and 20 h; Video S1: Animation images of microtomographic analysis of the internal microstructure of BT-PZT-F and BT-BF-F multiferroic composites archived in *wmv* files.

## Abbreviations

The following abbreviations are used in this manuscript:

BT	BaTiO_3_
PZT	Pb_0.94_Sr_0.06_(Zr_0.46_Ti_0.54_)_0.99_Cr_0.01_O_3_
F	Ni_0.64_Zn_0.36_Fe_2_O_4_ ferrite
BT	BiFeO_3_
EDS	Energy Dispersive Spectrometry
FESEM	Field Emission Scanning Electron Microscopy
SB	all electrons, both backscattered and secondary electrons, are detected (in FESEM research)
BSE	signal from the backscattered electron detector (in FESEM research)
RT	room temperature
*ρ*_DC_	DC resistivity
*σ*_DC_	DC conductance
*E*_a_	activation energy
*ε*	permittivity
tan*δ*	the dielectric loss tangent
*P-E*	ferroelectric hysteresis loop
*P*_r_	remanent polarization
*P*_m_	maximum polarization
*E*_c_	coercive field
*M*	magnetization
*M-H*	magnetic hysteresis loops
