# Supplementary material for: Study of the Microstructure and Ferroelectric and Magnetic Properties of Three-Component Multiferroic Composites
Source: Materials (Basel). 2026 Oct 13;19(20):4309. doi: 10.3390/ma19204309 (PMC13707007; doi:10.3390/ma19204309)
Supplement: Supplementary file 1 [file materials-19-04309-s001.zip › materials-4595981-supplementary.pdf]

Supplementary materials

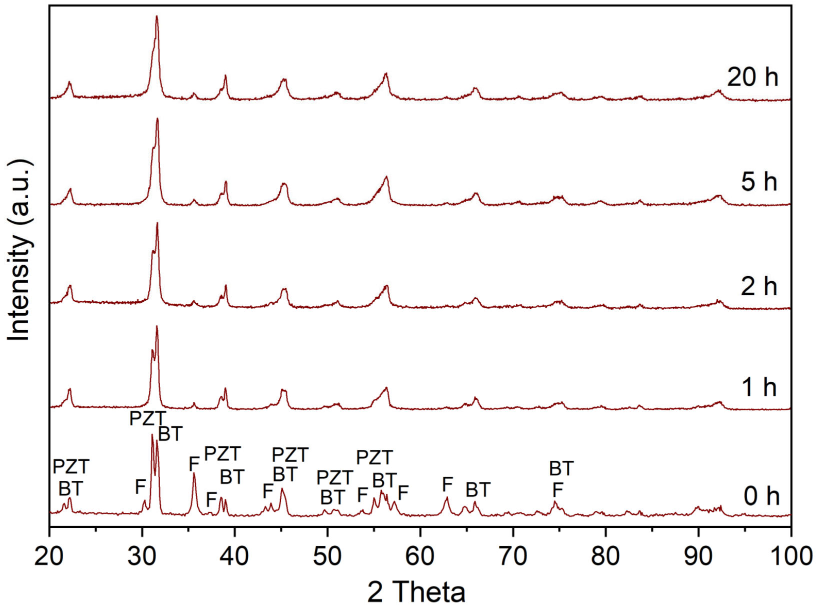

Figure S1 X-ray analysis of BT-PZT-F composite powder after the following mixing times: 1 h, 2 h, 5 h, 10 h, and 20 h.

Video S1 Animation images of microtomographic analysis of the internal microstructure of BT-PZT-F and BT-BF-F multiferroic composites archived in *wmv* files.
